# Psychometric properties of the fatigue questionnaire EORTC QLQ-FA12 and proposal of a cut-off value for young adults with cancer

**DOI:** 10.1186/s12955-018-0949-0

**Published:** 2018-06-15

**Authors:** Michael Friedrich, Erik Nowe, Dirk Hofmeister, Susanne Kuhnt, Katja Leuteritz, Annekathrin Sender, Yve Stöbel-Richter, Kristina Geue

**Affiliations:** 1 Department of Medical Psychology and Medical Sociology, University of Leipzig, Leipzig, Germany; 2 Medical Clinic II, Hospital St. Elisabeth and St. Barbara, Halle (Saale), Germany; 3 Faculty of Managerial and Cultural Studies, University of Applied Sciences Zittau/Goerlitz, Goerlitz, Germany

**Keywords:** Cancer-related fatigue, Young adults with cancer, Discriminant validity, Roc analysis, Psychometric properties

## Abstract

**Background:**

Young adult patients with cancer have to deal with their disease in an eventful phase of life. A common side effect of cancer and its treatment is cancer-related fatigue (CRF), a phenomenon which can thwart successful coping with developmental tasks. The aims of this study were to assess the psychometric properties of the EORTC QLQ-FA12, a new instrument for assessing physical, emotional and cognitive fatigue, in young adults with cancer, and to propose a cut-off value that indicates a need for further more specific diagnostics.

**Methods:**

In a sample of young adults who were first diagnosed with cancer between the ages of 18 and 39 years old, we assess the composite and item reliabilities as well as discriminant validity of the subscales for the EORTC QLQ-FA12. We also discuss two possible ways to calculate a summarizing score when conducting a receiver operating characteristic (ROC) analysis to find the cut-off value.

**Results:**

The EORTC QLQ-FA12 fit the sample (CFI = 0.96, SRMR = 0.04), had discriminant validity regarding its subscales and every subscale showed convergent validity (composite reliabilities were 0.92 for physical, 0.89 for emotional and 0.74 for cognitive fatigue). The sum of the first ten items with a range of 0 to 30 revealed a cut-off value of twelve or more with 91% sensitivity and 77% specificity.

**Conclusion:**

The new instrument EORTC QLQ-FA12 is able to distinguish between physical, emotional, and cognitive fatigue in young adult patients. It enables us to study different concepts of general fatigue without the need for additional items, and can be used as a screening instrument for young adults. Future research should investigate the multidimensional character of CRF.

**Electronic supplementary material:**

The online version of this article (10.1186/s12955-018-0949-0) contains supplementary material, which is available to authorized users.

## Background

The US National Cancer Institute defines adolescents and young adults (AYA) as a specific group of patients characterized by having been diagnosed with cancer between the ages of 15 and 39 [1]. The distinctive feature that AYA share is that they find themselves performing a balancing act: AYA are in a phase of life that is marked by change and accompanied by important and complex developmental tasks such as establishing financial and social independence, moving out of their parents’ home, and starting a career and a family [2]. At the same time AYA, have to deal with being ill with cancer as well as receiving treatments and follow-up care [3]. Even though the survival rates among AYA have stagnated for decades, the overall survival rate is about 80%. Combined with the increasing incidence rates of AYA cancer patients in Europe, Canada, and the USA [4], this is leading to a rising number of long-term survivors of young adulthood cancer.

Existing findings point out that cancer patients and survivors are greatly impacted by cancer-related fatigue (CRF) [5–7]. What’s more, it is a major problem for adolescents and young adults with cancer in particular [8]. CRF has been described in the scientific literature for more than 30 years as a significant side effect of cancer therapy with a psychological component [9, 10]. The National Comprehensive Cancer Network (NCCN) defined CRF as a multidimensional construct and, more precisely, as “a distressing, persistent, subjective sense of physical, emotional and/or cognitive tiredness or exhaustion related to cancer or cancer treatment that is not proportional to recent activity and interferes with usual functioning” [5]. With the image of an original and its reflection in mind, this definition describes forms of tiredness as *originals* of CRF and conforms to a multiple symptom concept. The multidimensionality regarding this concept refers to dimensions as expressions of *separate* symptoms [11]. The Fatigue Coalition, a multidisciplinary group of medical practitioners, researchers, and patient advocates [12], understands fatigue “as a multidimensional phenomenon, with physical, emotional, and cognitive manifestations” [13]. This suggests that the dimensions are indeed not expressions of several phenomena (e.g. physical, emotional, cognitive tiredness), but rather expressions of one and the same phenomenon, whereby the various forms of tiredness are different manifestations of the same underlying cause. Hence this definition describes forms of tiredness as *reflections* of CRF and can be understood as a multidimensional concept [11]. Accordingly they proposed a diagnostic interview guide for CRF [13] that is a set of diagnostic criteria for diagnosing CRF. This is described in more detail in the Additional file 1. The criteria are based on clinical experience, survey results, and discussions [12].

The question of whether the dimensions should be understood as separate phenomena (multiple-symptom concept) or as expressions of one and the same phenomenon (multidimensional concept) was recently discussed in a review, which, contrary to the consensus of experts, concluded that CRF should be considered a multiple-symptom concept [11].

CRF is not currently recognized as a mental disorder. It is not included in the Diagnostic and Statistical Manual of Mental Disorders, fifth edition (DSM-5) [14], and is not listed in the International Classification of Diseases, tenth revision (ICD-10) as an F-diagnosis (codes F00 to F99 describe mental and behavioral disorders). It is however listed in the clinical modification of the ICD-10 (ICD-10-CM) as code R53.0 (R00 to R99 describe symptoms that are not elsewhere classified) [15].

Cancer patients have reported experiencing CRF before, during, and after the acute therapy as well as multiple years after having completed treatment [16, 17]. CRF seems to impede daily life, social interactions, and physical activity [18–20]. Despite this, little research has been done to date on how CRF affects AYA. In a recent review done by Nowe et al. [21], only twelve studies on CRF in this age cohort were identified. Fatigue was found to be worse in AYA compared to both healthy controls and older cancer patients. Besides health status and age, gender seemed to also have an effect: women reported higher fatigue levels than men [21]. The vast majority of studies done since 1990 that have investigated CRF in AYA have not measured CRF with specific fatigue-questionnaires but rather with subscales of quality of life questionnaires or one-item scales [21]. Despite the consensus that a construct of CRF has to at least differentiate between a physical and a cognitive dimension [22], only two of the identified studies used the Multidimensional Fatigue Inventory (MFI) to detect the presence CRF [21]. The MFI-20 is probably the most commonly used CRF questionnaire in Europe, but the instrument appears to be less compatible with the diagnostic suggestions of the Fatigue Coalition. It consists of five subscales (four items each, response range 1 to 5): general fatigue, physical fatigue, mental fatigue, reduced motivation, and reduced activity. On the other hand, the European Organization for Research and Treatment of Cancer (EORTC) has developed the questionnaire module EORTC QLQ-FA12 [23], a new multidimensional instrument specifically for measuring CRF. The module that is described in more detail below assesses physical, emotional, and cognitive fatigue and as well as how they interfere with daily activities and social life. For both instruments (MFI-20 and QLQ-FA12), no total score is recommended, although the MFI-20 contains a subscale for measuring general fatigue. The items of this subscale cannot however be differentiated into physical, emotional, or cognitive dimensions. This would be possible using the subscales physical fatigue, reduced motivation, and mental fatigue. But the number of items of each subscale does not correspond to the number of physical, emotional, and cognitive diagnostic criteria that were proposed by Cella et al. [12]. Hence the QLQ-FA12 seems to be more suitable for investigating the three forms of tiredness separately, and for simultaneously screening for patients who could benefit from being given the clinical diagnostic interview that was proposed by the Fatigue Coalition.

With the objective of enabling an assessment of CRF in young adult cancer patients that can discriminate between physical, emotional, and cognitive fatigue based on the proposed diagnostic criteria, this study has three aims, of which the first two are necessary conditions for achieving the third (primary) aim:

1. to determine the psychometric properties of the EORTC QLQ-FA12 for young adult cancer patients,
2. to assess and compare two ways of calculating an overall fatigue score for the EORTC QLQ-FA12, and
3. to identify the cut-off point at which a patient should be considered for the proposed diagnostic interview.

## Methods

### Study participants

Participants were recruited for the prospective, longitudinal AYA-LE study [24] at 16 acute care hospitals, four rehabilitation clinics, and from two state tumor registries in Germany. In addition, other interested patients could register themselves via the internet or telephone. The baseline recruitment took place between May 2014 and December 2015. The study was approved by the Ethics Committee of the University of Leipzig (reference number 372–13-16,122,013).

Patients were included if: A) it was the first time they had been diagnosed with cancer; B) they were between 18 and 39 years old when they were diagnosed; and C) they had been diagnosed within the last four years. To avoid bias resulting from different treatment protocols, patients who were diagnosed before the age of 18 were not included, as younger patients in Germany are typically treated in pediatric oncology units. Patients that fulfilled these criteria were asked to fill out the questionnaire online or as hardcopy version twice. Our analysis is based on a sample *n* = 577 participants. Patients were excluded from the sample if they were not able to speak German, were physically or cognitively not able to participate, or did not provide written consent.

### Study measures

The sociodemographic characteristics we measured include: *age at time* of baseline interview, *age at diagnosis*, *time since diagnosis*, *educational degree,* and *sex*. Medical characteristics include *diagnosis* (ICD-10) and completed or ongoing *treatments* (chemotherapy, radiotherapy, surgery). Because there is a known connection between chemo- and radiotherapy and CRF [25], we also present the number of patients *who did not receive either* of these therapies. All data concerning socio-demographic and medical characteristics are based on self-reported information.

The *EORTC QLQ-FA12* (QLQ-FA12) is a new module of the Quality of Life Questionnaire Core 30 (QLQ-C30) developed by the EORTC group and intended to be used in conjunction with the QLQ-C30 [23]. The questionnaire core (QLQ-C30) and the module (QLQ-FA12) are translated into different languages and can be obtained for academic use free of charge at the EORTC Quality of life Group website [26]. The QLQ-FA12 consists of ten unidirectional items and two criteria variables, all of which range from 1 to 4 (higher values represent higher levels). The two criteria variables (fa11 and fa12) measure the extent to which fatigue interferes with daily activities (content of questions for role functioning) and social life (content of questions for social functioning). Hence, they measure the interference with two forms of usual functioning, like it is described in the definition of the NCCN. The ten items (fa1 to fa10) are assigned to three hypothetical subscales: physical (items fa1 to fa5), emotional (items fa6 to fa8), and cognitive fatigue (items fa9 and fa10). The scoring procedure follows that of the EORTC QLQ-C30, meaning that all scores are standardized to create a range of 0 to 100; no summary score has been suggested as of yet. Cronbach’s alpha of the three subscales ranges from 0.79 to 0.90 [23]. The former version (QLQ-FA13) of the questionnaire was published recently and contains the item wordings in the English language [27]. The item wordings in other languages can be obtained for free for academic use at the homepage of the EORTC group: http://groups.eortc.be/qol/why-do-we-need-modules.

To determine a cut-off point (the third aim of this study), two things are needed: a binary *reference standard* that indicates whether the outcome is positive or negative, and a *test (score)* that predicts the target conditions. For the test, we used the first ten items of the FA12 that are assigned to the three subscales. To create the reference standard, we used a total of thirteen individual items from four different instruments (EORTC QLQ-C30, EORTC QLQ-FA13, HADS and SCNS SF-34). These instruments contained items suitable in content for indicating the target condition whereby it is recommendable for a patient to be given the diagnostic interview proposed by the Fatigue Coalition [13]. Table 1 presents the thirteen items that were selected from the four instruments. A more detailed description of the items and their assignment to the diagnostic criteria is given in the Additional file 1 in Table S1.

**Table 1:** Items assigned to the diagnostic criteria

Instrument (Description, References)	*EORTC QLQ-C30 (containing 30 items)* (Quality of Life Questionnaire Core of the EORTC group, [42, 43])
Measured trait	health related quality of life and symptoms in cancer patients
Selected items (corresponding scale)	c10, c12, c18 ([physical] fatigue); c20, c25 (cognitive functioning); c24 (emotional functioning); c3 (physical functioning); c11 (symptom item insomnia)
Instrument (Description, References)	*EORTC QLQ-FA13 (containing 13 items)* (Phase III (former) fatigue module of the EORTC group, [27])
Measured trait	cancer-related fatigue
Selected items (corresponding scale)	fa13_11 (dropped in QLQ-FA12); fa13_12 (criteria variable daily activities, labeled as item fa11 in QLQ-FA12)
Instrument (Description, References)	*HADS (containing 14 items)* (Hospital Anxiety and Depression Scale, [44, 45])
Measured trait	anxiety and depression in physically impeded patients
Selected items (corresponding scale)	ha1, ha6 (subscale anxiety)
Instrument (Description, References)	*SCNS SF-34 (containing 34 items) *(Supportive Care Needs Survey Short-Form, [46–48])
Measured trait	perceived supportive care needs
Selected items (corresponding scale)	s2 (subscale physical and daily living need)

### Statistical analyses

Statistical analyses were done with IBM SPSS Statistics 23, IBM SPSS AMOS 23, and Microsoft EXCEL 2010. Missing values were estimated on the item level using the Expectation Maximization (EM) algorithm [28] that is implemented in SPSS. Imputed values that exceeded the possible range were set to the nearest possible value.

### Aims 1) and 2) psychometric evaluation and overall fatigue measure

The psychometric evaluation of the EORTC QLQ-FA12 for young adults with cancer comprises confirmatory factor analyses of the following models:

M1) the First-Order three-factorial FA12-Model, conceptualized by Weis et al. (p.6, figure 2),

M2) a Second-Order factor model (General Fatigue *Score*)

M3) a First-Order one-factorial model (General Fatigue *Index*)

*Model M1 (EORTC QLQ-FA12)* represents the measurement model as it is intended by the developers of the questionnaire. Hence the two criteria variables have to be present in this model. Even if they conceptually do not contribute to any of the fatigue scores, they measure the interference of the three forms of fatigue with two forms of usual functioning. For this model, we investigate the following psychometric properties (aim 1): model fit, composite reliability (CR), item reliabilities (squared multiple correlations, SMC), and discriminant validity using the Fornell-Larcker-Criterion [29], which is based on a comparison of average variance extracted (AVE) and the squared correlations between the domains. CR measures the amount of variance of the items that is bound by their common factor. If CR shows a value greater than 0.6, it is considered adequate [30]. A conservative lower bound for CR is Cronbach’s Alpha, which is also presented. SMC measures the amount of the item’s variance that is explained by the respective latent factor. No rule of thumb for adequate item reliability can be suggested, but the SMC should be smaller than the CR [30]. Furthermore, the items should share on average more than 50% of their variance with their composite. As such a value of AVE greater than 0.5 is passable [29, 30]. Two domains (say D1 and D2) have discriminant validity if they are statistically distinguishable. This is formally satisfied if the AVE of every domain is higher than their squared correlation r^2^. That means if both of the following equations work out [29]:

$$ AV{E}_{D1}>{r}_{\left(D1,D2\right)}^2\kern0.5em and\kern0.5em AV{E}_{D2}>{r}_{\left(D1,D2\right)}^2 $$

The formulas to compute the scores for each domain are presented in the Additional file 1 in section “*Model M1*”.

Models M2 (separated domains) and M3 (not separated domains) serve to discuss two different conceptualizations of an overall fatigue measure that is based on the ten single items of the FA12 (aim 2). The two criteria variables 11 and 12 are excluded from both models, because they do not measure fatigue, but rather the extent to which it interferes with daily life.

*Model M2 (General Fatigue Score)* takes into account the fact that the items belong to different domains and assumes general fatigue to be a quantity that is *constituted by the three components* (physical, emotional and cognitive fatigue) equally. This multidimensional model reflects a three dimensional concept of general fatigue. Because the components contribute equally they can compensate for each other and it is of no concern which type of fatigue causes the burden. Patients who are complaining about all of the symptoms of only one dimension end up having the same score, regardless of which dimension is in question. A numerical example is presented in the Additional file 1. Acceptable fit of this model would give statistical justification for using *a score that is composed of the three domains* as a measure for general fatigue. The formula to compute the overall score is presented in the Additional file 1 in section “*Model M2*”.

*Model M3 (General Fatigue Index)* leaves out the information that the items belong to different domains, implying that all items measure the same quantity. This one-dimensional model assumes general fatigue as it is *constituted by the ten items*, regardless of which component the item belongs to. Patients who are complaining about all of the symptoms in only one dimension end up with different scores, depending on the dimension, because the dimensions have different numbers of symptoms. A numerical example is presented in the Additional file 1. M3 models the score we work with, when we are simply summing the ten items, or giving every domain a different weight corresponding to its number of items. A different number of items -even if only in one domain- would change the concept. One could say that the number of items from each component weights the components contribution to a one dimensional concept of general fatigue. That means that physical fatigue is a more burdensome form of fatigue than emotional fatigue, and emotional fatigue is more important than cognitive fatigue, because the former has fewer items than the latter. That is also an implication of the concept underlying the diagnostic criteria of the Fatigue Coalition. Acceptable fit of this model would give statistical support for using *a score that is composed of the ten items* as a measure for general fatigue. This formula is presented in the Additional file 1 in section “*Model M3*”.

To *judge model fit,* we used a combinational rule of the CFI (comparative fit index) and the SRMR (standardized root mean square residual) [31]. Models are rejected if both CFI and SRMR indicate poor fit (CFI < 0.95 and SRMR> 0.06). For comparability of our results, we also present the TLI (Tucker-Lewis-Index), the RMSEA (Root Mean Square Error of Approximation) including its 90% confidence interval, and the AIC (Akaike’s Information Criterion).

### Aim 3) ROC analysis

The ROC analysis was done on a subsample of *n* = 548 patients who did not report a comorbid depression, because the symptoms should not primarily be the consequence of comorbid psychic disorders (sixth condition of diagnosis). The information collected on comorbid depression came from the answers to the open question “*At the present, do you additionally suffer from a serious physical or psychological disease and if yes, from what?”*

Because there is no gold standard but only a proposal of diagnostic criteria by members of the Fatigue Coalition [12, 13], one could use a statistical approach to differentiate between respondents with and without fatigue and identify a cutoff, e.g. at the 75th percentile [32, 33]. We decided however to use a more theory-based statistical approach to avoid some of the arbitrariness that comes with a non-theoretical approach. To do this, we assign thirteen individual items that correspond best to the CRF diagnosis criteria proposed by the Fatigue Coalition. The criteria are summarized in the Additional file 1, as well as the construction of the binary reference standard and the justification for interpretation of the ROC analysis results.

The test that predicts the conditions of the standard could be calculated in line with either the M2 or M3 model. We have to use the M3 model despite the acceptability of its model fit, because it is closer to the composition of the diagnosis criteria and because the reliability for predicting the reference standard is of more concern than the reliability for measuring one common quality. All criteria symptoms are added up to one value, regardless of whether the symptom is of a physical, emotional, or cognitive nature. To make the test easy to employ, we refrain from the usual standardization of the range from 0 to 100 and used the sum of the ten items as if each were coded from 0 to 3. For items ranging from 1 to 4 the formula is:

$$ test= sum\left( fa1,\dots, fa10\right)-10 $$

To characterize the ROC Analysis, we present the area under curve (AUC) that corresponds to the detectability of the signal or, in other words, to the probability that the test can correctly identify the conditions of the standard [34]. More importantly, we also present cut-off values along with the following coefficients:

- Sensitivity (SEN, ratio of true positive predictions to all positive conditions)
- Specificity (SPE, ratio of true negative predictions to all negative conditions)
- Youden Index (J, diagnostic ability, difference between true positive rate (SEN) and false positive rate (1-SPE) [35, 36])
- Positive predicted value (PPV or precision, ratio of true positive predictions to all positive predictions)
- Negative predicted value (NPV, ratio of true negative predictions to all negative predictions)
- Accuracy (ACC, ratio of correct predictions to all predictions of the conditions of the standard variable).

## Results

All of the *n* = 577 young adults with cancer we surveyed completed the questionnaire. We estimated the missing values for 91 items (from the instruments mentioned above in section *Study measures*). They ranged from 0 (0%) to 16 (2.8%) per item and from 0 (0%) to 34 (37.4%) per patient. Less than 170 (0.3%) missing values were imputed (170 missing values within 52,507 values, while using values for imputation from 91 items multiplied by 577 cases).

A group of *n* = 29 patients who reported a comorbid depression were excluded from the ROC analysis. The excluded patients were mostly women (93% vs. 72% in the analyzed sample) who had been diagnosed with Hodgkin lymphoma (31% vs. 16%) or gastrointestinal cancer (17% vs. 4%) within the previous two months (7% vs. 1%). The comparisons of these percentages were significantly different with type-I-error probability p below 0.05.

### Characteristics of the sample

Table 2 presents the sample characteristics for the whole sample (*n* = 577). The average age at diagnosis was 29 (range from 18 to < 40 years). The average time since diagnosis was nearly one year (11.9 months, range, 1 month to 3.7 years). Mean age at baseline was 30 (range: 18 to 42). Women did make up 73% of the sample, and about two thirds (68%) of the patients were at least 26 years old.

**Table 2:** Sociodemographic and medical characteristics of the sample (*n* = 577)

Sociodemographic characteristics	*N* (%)
Sex	577 (100.0)
Male	153 (26.5)
Female	424 (73.5)
Age at Interview (M = 30.3, SD = 6.1)	577 (100.0)
18 to < 26 years	164 (28.4)
26 to < 42 years	413 (71.6)
Education	573 (100.0)
No educational degree/student	6 (1.0)
Basic educational degree (< 10 years)	37 (6.5)
Secondary educational degree (10 years)	190 (33.2)
Highschool degree (> 10 years)	340 (59.3)
Medical characteristics	N (%)
Age at Diagnosis (M = 29.3, SD = 6.1)	577 (100.0)
18 to < 26 years	184 (31.9)
26 to < 40 years	393 (68.1)
Time since Diagnosis (M = 11.9, SD = 8.0)	577 (100.0)
up to 2 months	8 (1.4)
> 2 to 4 months	38 (6.6)
> 4 to 6 months	69 (12.0)
> 6 to 12 months	269 (46.6)
> 12 to 24 months	145 (25.1)
more than 24 months	48 (8.3)
Diagnosis	577 (100.0)
Breast Cancer [C50]	150 (26.0)
Hodgkin Lymphoma [C81]	99 (17.2)
Gynecological Cancer [C51-C57]	51 (8.8)
Testicular Cancer [C62]	50 (8.7)
Non-Hodgkin Lymphoma [C82-C90]	42 (7.3)
Haematological Cancer [C91-C95]	38 (6.6)
Thyroid Cancer [C73]	32 (5.5)
Gastrointestinal Cancer [C15-C26]	29 (5.0)
Sarcoma [C40-C41, C46-C49]	26 (4.5)
Melanoma [C43]	19 (3.3)
Other C, D00-D48 and Carcinoma in situ	41 (7.1)
Therapies^a, b^	577 (100.0)
Chemotherapy^c^	443 (76.8)
Radiotherapy^d^	264 (45.8)
Surgery	427 (74.0)
Neither chemo- nor radiotherapy	85 (14.7)

^a^multiple answers possible; ^b^ Due to further validation of data there are deviations to the baseline medical therapies published in the study protocol [49].; ^c^ including Radio-Chemotherapy; ^d^ including nuclear therapies and Radio-Chemotherapy

### Aim 1) psychometric properties

**Fig. 1:**
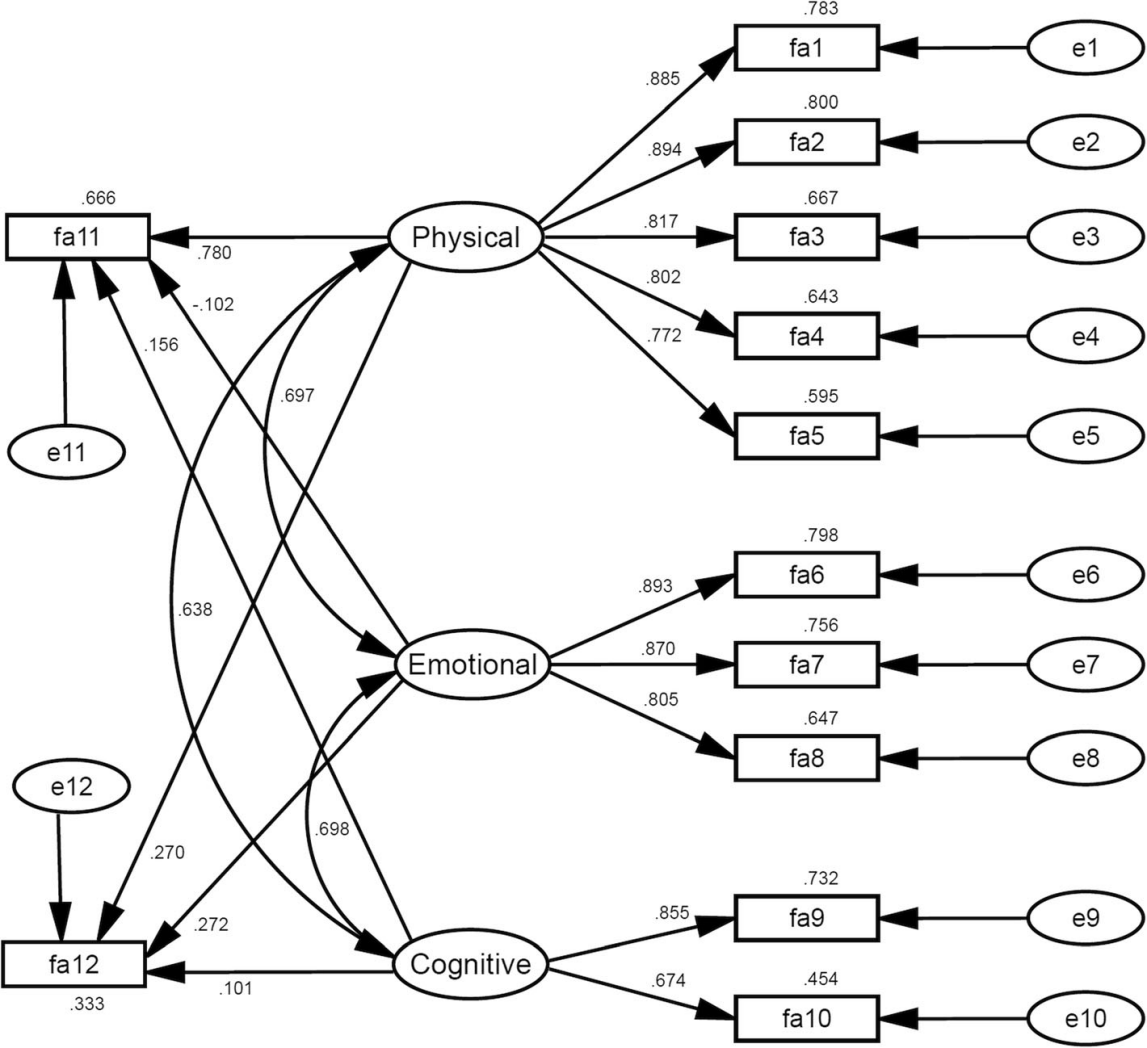
Model 1 (EORTC QLQ-FA12 Model). *Linear arrows* show standardized regression weights. *Curved arrows* show correlations. Values above or under (variable fa12) the r*ectangles* show squared multiple correlations (SMC, item reliabilities for fa1 to fa10, explained variance for variables fa11 and fa12). Variables fa11 and fa12 correspond to items fa12 and fa13 of the former version EORTC QLQ-FA13 in this order

Figure 1 presents the psychometric properties of the fatigue questionnaire EORTC QLQ-FA12 (model M1). The model fitted the sample (CFI = 0.96 and SRMR = 0.04, Table 3). Item reliabilities (SMC) ranged from 0.60 to 0.80 for the physical fatigue scale, from 0.65 to 0.80 for the emotional fatigue scale, and from 0.45 to 0.73 for the cognitive fatigue scale. The correlations between the three scales ranged from 0.63 to 0.70.

**Table 3:** Model Fit (*n* = 577)

Model-Fit		Chi^2^(df)	p	Chi^2^/df	CFI	TLI	SRMR	RMSEA (90%-CI)	AIC
M1.	EORTC-FA12 Model	266.2 (47)	< 0.001	5.7	**0.955**	0.973	**0.042**	0.09 (0.08–0.10)	352.2
M2.	General Fatigue Score	180.2 (32)	< 0.001	5.6	**0.963**	0.948	**0.045**	0.09 (0.08–0.10)	246.3
M3.	General Fatigue Index	837.9 (35)	< 0.001	23.9	0.800	0.743	0.091	0.20 (0.19–0.21)	897.9

CFI (comparative fit index), TLI (Tucker-Lewis-Index), SRMR (standardized root mean square residual), RMSEA (Root Mean Square Error of Approximation), AIC (Akaike’s Information Criterion). Bold values indicate acceptable fit (CFI ≥ 0.95 or SRMR≤0.06)

The interference of the three subscales with daily activities (fa11) and with social life (fa12) differed. Physical fatigue was the major predictor for fa11 (standardized regression weight w = 0.780, *p* < 0.001), besides cognitive (w = 0.156, *p* = 0.002) and emotional fatigue (w = − 0.102, *p* = 0.043). For fa12 we found that that physical (w = 0.270, *p* < 0.001) and emotional fatigue (w = 0.272, *p* < 0.001) interfered with social life significantly, but the effect of cognitive fatigue (w = 0.101, *p* = 0.126) did not.

The composite reliabilities were 0.92 for physical fatigue, 0.89 for emotional, and 0.74 for cognitive fatigue (Table 4, column CR). CR was greater than 0.6 and greater than their corresponding SMCs for every scale. On average the three composites extracted 70% (physical fatigue), 73% (emotional fatigue), and 59% (cognitive fatigue) of the variance within their corresponding items (Table 4, bold values on the diagonal).

**Table 4:** Discriminant and convergent validity for model M1 (n = 577)

Domain	Physical	Emotional	Cognitive	CR	Cronbach’s Alpha^a^
Physical fatigue	**0.698**	0.487	0.407	0.920	0.918
Emotional fatigue	0.697	**0.734**	0.487	0.892	0.891
Cognitive fatigue	0.638	0.698	**0.593**	0.742	0.732

*Above the diagonal (underlined values)*: squared correlations (r^2^). *On the diagonal (bold values)*: average variance extracted (AVE). *Below the diagonal*: correlations (r). Discriminant validity is indicated if AVE_D1_ > r^2^_(D1,D2)_ and AVE_D2_ > r^2^_(D1,D2)_ and convergent validity if composite reliability CR > 0.6.; ^a^ based on standardized items

All composites were statistically distinguishable and had discriminant validity, because in every case the shared variance between two domains was smaller than the AVE of the two domains (e.g. physical and emotional fatigue: r^2^(physical, emotional) = 0.49 was smaller than AVE(physical) = 0.70 and smaller than AVE(emotional) = 0.73 (Table 4, AVE: bold values on the diagonal, r^2^: underlined values above the diagonal).

### Aim 2) overall fatigue measure

The fit of both models is shown in Table 3. The second-order factor model M2 presents the three domains as composing first-order composites for general fatigue. Model fit was acceptable (CFI = 0.96 and SRMR = 0.05). The CR for general fatigue was 0.87 and the AVE was 0.68; SMCs are 0.63 (physical domain), 0.77 (emotional domain), and 0.65 (cognitive domain). For the domains, the values of CR/AVE were 0.92/0.70 (physical), 0.89/0.73 (emotional), and 0.74/0.59 (cognitive). The first-order factor model M3 presents the items as composing general fatigue, without differentiating between the components that the items correspond to. The CR for general fatigue was 0.92 and the AVE was 0.54; SMCs ranged from 0.22 (item fa10) to 0.73 (item fa1). While these coefficients showed acceptable characteristics, the fit for this model was not acceptable (CFI = 0.80 and SRMR = 0.09).

### Aim 3) ROC analysis

Table 5 presents the results of the ROC analysis. Two cut-off values (≥11 and ≥ 12) had sufficient sensitivity and specificity (SEN ≥ 90 and SPE ≥ 70) and the cut-off value of ≥12 had the higher sum of SEN and SPE. It showed the following characteristics:

**Table 5:** ROC analysis (*n* = 548)

Cutoff (case ≥ ...)	Value (95% CI)					
	SEN	SPE	Youden J	PPV	NPV	ACC
8	98 (96–100)	53 (48–57)	0.51 (0.48–0.53)	37 (31–42)	99 (98–100)	63 (59–67)
9	97 (93–100)	60 (55–65)	0.57 (0.53–0.59)	40 (34–46)	98 (97–100)	68 (64–72)
10	94 (90–98)	66 (61–70)	0.60 (0.56–0.62)	43 (37–49)	98 (96–99)	72 (68–75)
11	92 (88–97)	71 (67–75)	0.64 (0.60–0.66)	47 (41–53)	97 (95–99)	76 (72–79)
12	**91 (86–96)**	**77 (73–81)**	**0.68 (0.64–0.70)**	**52 (45–59)**	**97 (95–99)**	**80 (77–83)**
13	89 (83–95)	81 (77–84)	0.70 (0.66–0.73)	56 (49–63)	96 (94–98)	82 (79–86)
14	83 (76–90)	83 (79–86)	0.66 (0.62–0.69)	57 (50–65)	95 (92–97)	83 (80–86)
15	81 (74–88)	86 (83–90)	0.67 (0.63–0.71)	62 (55–70)	94 (92–96)	85 (82–88)
16	72 (64–80)	89 (86–92)	0.62 (0.57–0.65)	65 (57–73)	92 (89–95)	86 (83–89)
17	62 (53–71)	92 (89–94)	0.54 (0.49–0.58)	67 (59–76)	90 (87–93)	85 (82–88)
18	52 (43–61)	94 (92–96)	0.46 (0.41–0.51)	70 (61–80)	88 (85–91)	85 (82–88)

Cut-off values for the general fatigue index (0–30). SEN (sensitivity) SPE (specificity), PPV (positive predicted value), NPV (negative predicted value), ACC (accuracy). Values with SEN or SPE below 50% are not presented

Of all of the patients with the positive condition, 9 out of 100 were missed (SEN = 91, 95%-CI: 86–96). Of all of the patients with the negative condition, 23 out of 100 were referred for further diagnostics nonetheless (SPE = 77, 95%-CI: 73–81). The difference between the true positive rate minus the false positive rate was 68 percentage points (Youden J_≥12_ = 0.68, 95%-CI: 0.64–0.70). Out of 100 positive predictions, nearly 50 were correct (PPV = 52, 95%-CI: 45–59) and out of 100 negative predictions, only 3 were incorrect (NPV = 97, 95%-CI: 95–99). Altogether, 4 of 5 predictions were correct (ACC = 80, 95%-CI: 77–83). According to classification guidelines proposed by Zhu et al. [37], the detectability of general fatigue by this test was excellent: AUC = 0.91 (95%-CI, 0.88–0.94).

## Discussion

### Aim 1) psychometric evaluation

The EORTC-FA12 fatigue module shows sufficient psychometric properties. This suggests convergent validity and discriminant validity in this specific age-cohort of cancer patients. In other words: this statistically justifies using this instrument among young adults with cancer.

Regarding the criteria variables we could replicate the results from the original study of Weis et al. [23] for physical fatigue. Emotional fatigue showed similar interference with social life, but its interference with daily activities pointed into the opposite direction. For cognitive fatigue we found an effect on daily activities, while the original study did not and we found no significant effect on social life, while the original study did find an effect. It is reasonable to assume that these differences are due to our special sample of patients. AYA have a different social life and different daily activities than older patients. This is what makes this group of patients special and it can explain these differences.

### Aim 2) two concepts of an overall fatigue measure

We pointed out above that there are two fundamental dissimilar definitions of CRF (NCCN: tiredness as *original* vs. Fatigue Coalition: tiredness as *reflection*). Then we found that the dissimilarity shows itself in the disagreement about what CRF is, e.g.: Is it a mental disorder (DSM-5) or merely a symptom (ICD-10)? Should it be conceptualized as a multiple symptom concept or as a multidimensional concept? Should it be modeled as a second-order factor model (M2) or as a first-order one-factorial model (M3)? While we investigated the last question, our results indicate that model M2 is the statistically sound conceptualization of general fatigue and model M3 is not. Therefore physical, emotional, and cognitive fatigue might be separate phenomena, a conclusion that is in line with other studies’ findings [11]. Although this does not yet suffice for justifying the multiple symptom concept, it does reveal a conceptual discrepancy that impedes progress in CRF research. To decide how CRF should be understood, the consensus of experts might not be sufficient. Moreover, it seems imperative to elucidate the pathogeneses of the separate phenomena [11]: e.g. Have they different pathogeneses or not? Are there factors that affect one form of fatigue but not the other? Do the forms of fatigue behave differently? Could it be possible that one form of fatigue can lead to another and, if so, could they develop a cycle that can exist independently from the presence of the first trigger? Despite the answers to these questions, the next step towards progress in CRF research requires that clear distinctions be made between physical, emotional, and cognitive fatigue.

### Aim 3) proposed cut-off value

We conducted a ROC analysis with a reference standard that is based on the proposed ICD-10 criteria for diagnosing CRF. Even though this standard is just an approximation of the diagnostic criteria, it represents a useful tool for limiting the candidates for diagnostic interviews in a way that is backed up by theoretical considerations. We also know about its limitations: Regarding the true positive condition we do not know if a single patient:

- experiences several hours of persistent post-exertional malaise (symptom A11, no item(s) assigned),
- has all the named symptoms within the same two weeks of the past month (different timeframes of the items),
- can attribute them to feeling fatigued,
- suffers from clinically significant distress or impairments in important areas of functioning
- has a history with evidence that the symptoms are a consequence of cancer or its therapy (even though all of the participants in our sample were diagnosed with and treated for cancer)
- or has additional psychiatric comorbidities besides depression (participants, who reported a depression as comorbidity, were excluded in this analysis).

On the other hand, if a patient does not to have fatigue according to these criteria, we can be more confident that this is accurate, because with a sum of three or less, a patient cannot meet five or more of the ten symptoms and is therefore unlikely to receive a positive diagnosis. Patients with a sum of three could meet four symptoms if they additionally met symptom A11, which has no corresponding item(s) in our approximation. But even if they did fulfill A11 as well as the conditions named above, a patient could not receive a positive diagnosis. Consequently, this standard identifies nominees for the proposed diagnostic interview; but does not represent a diagnosis in and of itself.

### Clinical implications

Balancing between two fundamentally different conceptualization of CRF, our findings indicate a multiple-symptom concept of CRF. We recommend observing physical, emotional, and cognitive fatigue separately. An overall score can be an addition and should be calculated in line with model M2 using these three dimensions.

Taking into consideration that we chose a diagnostic criteria proposal, the cut-off value shows reliable characteristics but is not in line with the recommended overall score. Furthermore, the cutoff cannot replace a clinically justified diagnosis of CRF. It can merely pre-select patients who should undergo the proposed clinical diagnostic interview.

To date, we are not able to propose cut-off values for physical, emotional, or cognitive fatigue separately because no clinical diagnostic criteria exist yet to even approximate a standard for diagnosing these forms of fatigue.

### Limitations

We estimated missing values with the EM algorithm, which does not consider an additional share of error for the missing values. Therefore standard errors are smaller; confidence intervals narrower, and respectively the *p* values (type-I-error probabilities) are smaller. The bias on account of this procedure is expected to be small, and most techniques for handling missing data are expected to yield similar results because proportions of missing values were below 5 % [38, 39]. Additionally, we conducted the ROC analysis using an approximation of the diagnostic criteria based on self-reported items that are close to the criteria. Hence the results are biased in three different ways. There is bias due to approximation (1) that we tried to minimize as best as possible (see Additional file 1: Table S1). Then there is bias due to self-report (2). Because CRF is a symptom that is *perceived by the patient* [5], it seems to be the most precise possible to rely on the self-report of the patient. Accordingly it seems that a clinical judgment could be a source of bias too, still there is no theoretical *and* statistical sound definition of CRF that could avoid bias in clinical judgement. In addition there is bias that is connected to halo effects (3). It originates from items that are located near to the items of the test, when they are read before self-reporting the actual answer. However, this type of bias is contained in our study too, because our main focus was not to avoid it but to use the questionnaire as it is recommended by the EORTC. Furthermore, women comprise the majority of our sample. Although this is to be expected (German national prevalence estimates show 61% of cancer patients aged 0 to 44 are women [40]), with 74% of the sample being female the generalizability of our results regarding sex is somewhat biased. For instance, they might be biased regarding effects that are related to depression, because depression is more common among female patients [41].

## Conclusions

The new EORTC Quality of Life Module for measuring cancer-related fatigue (EORTC QLQ-FA12) is a very promising instrument for intensifying the research on CRF in young adult patients. This instrument

- is statistically valid and can discriminate between physical, emotional, and cognitive fatigue;
- provides an overall measure of CRF that is in line with the definition of the NCCN;
- and can be used as a screening instrument to identify patients who could benefit from the clinical diagnostic interview proposed by the Fatigue Coalition.

## Additional file

Additional file 1: Supplementary material with comments on the tested models and on the ROC analysis. (DOCX 1761 kb)

## Abbreviations

ACC: Accuracy
AIC: Akaike’s Information Criterion
AUC: Area under curve
AVE: Average variance extracted;
AYA: Adolescent and young adults
CFI: Comparative fit index
Chi^2^: Chi squared statistic
CI: Confidence interval
CR: Composite reliability
CRF: Cancer-related fatigue
df: Degrees of freedom
DSM-5: Diagnostic and Statistical Manual of Mental Disorders, fifth edition
EM: Expectation maximization
EORTC: European Organization for Research and Treatment of Cancer
FA12: Quality of Life Questionnaire fatigue module 12 items
FA13: Quality of Life Questionnaire fatigue module 13 items (former version of FA12)
HADS: Hospital Anxiety and Depression Scale
ICD-10 CM: International Classification of Diseases, tenth revision, clinical modification
J: Youden index
M: mean
MFI-20: Multidimensional Fatigue Inventory 20 items
NCCN: National Comprehensive Cancer Network
NPV: Negative predicted value
p: Type-I-error probability
PPV: Positive predicted value
QLQ-C30: Quality of Life Questionnaire Core 30 items
RMSEA: Root mean square error of approximation
ROC: Receiver operating characteristic
SCNS SF-34: Supportive Care Needs Short Form 34 items
SD: Standard deviation
SEN: Sensitivity
SMC: Squared multiple correlations
SPE: Specificity
TLI: Tucker-Lewis-Index
