# Supplementary material for: Psychometric properties of the fatigue questionnaire EORTC QLQ-FA12 and proposal of a cut-off value for young adults with cancer
Source: Health Qual Life Outcomes. 2018 Jun 15;16:125. doi: 10.1186/s12955-018-0949-0 (PMC6002999; doi:10.1186/s12955-018-0949-0)
Supplement: Supplementary file 1 — Supplementary material with comments on the tested models and on the ROC analysis. (DOCX 1761 kb) [file 12955_2018_949_MOESM1_ESM.docx]

Additional file 1

*Psychometric properties of the fatigue questionnaire EORTC QLQ-FA12 and proposal of a cut-off value for young adults with cancer*

Michael Friedrich^1*^, Erik Nowe^1^, Dirk Hofmeister^1^, Susanne Kuhnt^1^, Katja Leuteritz^1^, Annekathrin Sender^1^,
Yve Stöbel-Richter^2^, Kristina Geue^1^

**Address**:
^1^ Department of Medical Psychology and Medical Sociology, University of Leipzig, Leipzig, Germany
^2^ Faculty of Managerial and Cultural Studies, University of Applied Sciences Zittau/ Goerlitz, Goerlitz, Germany

^*^ Corresponding author

Comments on the tested models

Model M1: EORTC QLQ-FA12 (psychometric evaluation of the intended first-order-factor model)

This multidimensional model (see figure 1 in the main document) represents the three components of fatigue (physical, emotional and cognitive fatigue) as coexisting types of fatigue. Their similarities are modeled as correlations between them, assuming that they do not compose one common quality. Nevertheless it would lead to exactly the same results, if we modeled one common second-order factor instead of the correlations.

The special feature of model M1 is the inclusion of the two criteria variables fa11 and fa12, as it is intended by the developers of the questionnaire [1]. The regression weights directed from the fatigue components to them are not meant as cross loadings. Therefore these variables are not located aligned with the other items fa1 to fa10. Items fa11 and fa12 represent the interference of CRF with daily life (fa11: daily activities, fa12: social life) and are therefore not relevant for measuring fatigue itself.

**Scoring information**: Model M1 does not imply the use of an overall score. Instead it expresses only the use of scores for each hypothetical component. The scoring procedure for the EORTC QLQ-FA12 follows the scoring for the EORTC QLQ-C30 [2], that is, all scores are computed as means of their items and are standardized to a range from 0 to 100.

**Example 1:**  For items that range from 1 to 4, the scores for each subscale are calculated using the formula:

$$score=\left( mean\left( items \right)-1 \right)\cdot\frac{100}{3}$$

$$physical fatigue=\left[ \frac{1}{5}\left( fa1+fa2+fa3+fa4+fa5 \right)-1 \right]\cdot\frac{100}{3}$$

$$physical fatigue=\left[ \frac{1}{3}\left( fa6+fa7+fa8 \right)-1 \right]\cdot\frac{100}{3}$$

$$physical fatigue=\left[ \frac{1}{2}\left( fa9+fa10 \right)-1 \right]\cdot\frac{100}{3}$$

For instance, a patient, who answers on each physical item with 3 (= “quite a bit”) reaches a physical fatigue score of 66.7.

Model M2: General Fatigue Score (second-order factor model: separated domains)


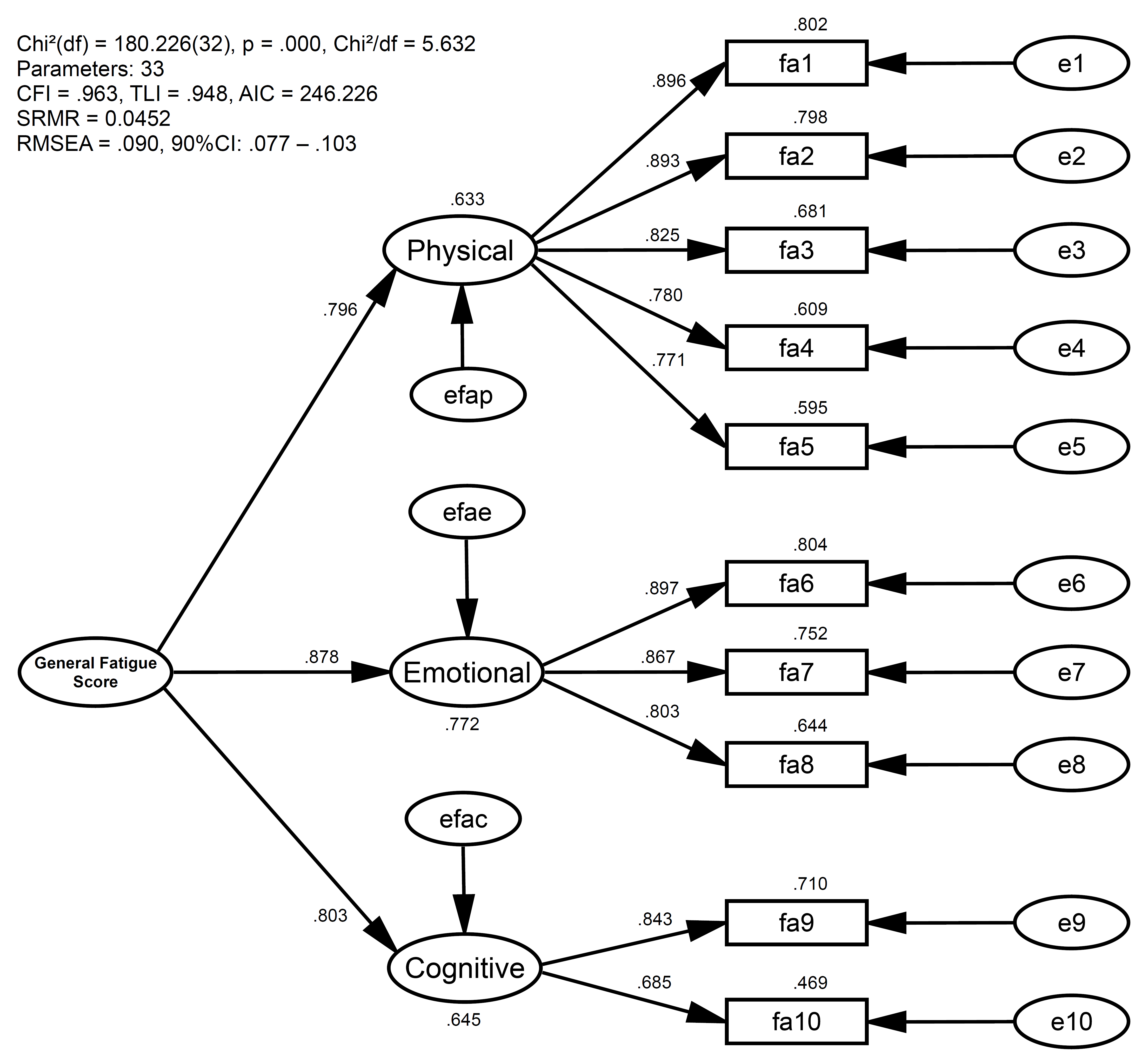


**Supplementary figure F1**: Model M2, second-order factor model, without criteria variables.

This model represents the three components of fatigue (physical, emotional and cognitive fatigue) as coexisting types of fatigue that are equally composing one common quality (general fatigue).

**Scoring information**: The model implies the use of scores for the three hypothetical components (physical, emotional and cognitive fatigue) as it was described above for model M1. Additionally, it implies the use of an overall score that is composed of the three components. The overall score is calculated using the following formula:

$$Overall score= \left( mean\left( mean\left( physical items \right),mean\left( emotional items \right), mean\left( cognitive items \right) \right)-1 \right) \cdot\frac{100}{3}$$

$$General fatigue score= \left[ \frac{1}{3}\left( \frac{1}{5}\left( fa1+fa2+fa3+fa4+fa5 \right)+\frac{1}{3}\left( fa6+fa7+fa8 \right)+\frac{1}{2}\left( fa9+fa10 \right) \right)-1 \right]\cdot\frac{100}{3}$$

**Example 2:** Consider Patient A, who answers each physical item with 3 (= “quite a bit”) and all other items with 1 (=”not at all”). S/he would reach a value of 22.2. Another participant, patient B, who answers all cognitive items with 3 and all other items with 1, ends up with the same score:

$$\boldsymbol{Patient A}= \left[ \frac{1}{3}\left( \frac{1}{5}(3+3+3+3+3)+\frac{1}{3}(1+1+1)+\frac{1}{2}(1+1) \right)-1 \right]\cdot\frac{100}{3}=22.2$$

$$\boldsymbol{Patient B}= \left[ \frac{1}{3}\left( \frac{1}{5}(1+1+1+1+1)+\frac{1}{3}(1+1+1)+\frac{1}{2}(3+3) \right)-1 \right]\cdot\frac{100}{3}=22.2$$

Note that M2 treats the *domains* *equally* and the items are treated equally only within their own domain. This means the *items are treated differently* within the score: The weight of the item depends on the domain to which the item corresponds:

General fatigue $= \left[ \frac{1}{3}\left( \frac{1}{5}\left( fa1+fa2+fa3+fa4+fa5 \right)+\frac{1}{3}\left( fa6+fa7+fa8 \right)+\frac{1}{2}\left( fa9+fa10 \right) \right)-1 \right]\cdot\frac{100}{3}$

$= \left[ \left( \frac{1}{3}\cdot\frac{1}{5}\left( fa1+fa2+fa3+fa4+fa5 \right)+ \frac{1}{3}\cdot\frac{1}{3}\left( fa6+fa7+fa8 \right)+ \frac{1}{3}\cdot\frac{1}{2}\left( fa9+fa10 \right) \right)-1 \right] \cdot\frac{100}{3}$

$= \left[ \left( \frac{1}{15}\left( fa1+fa2+fa3+fa4+fa5 \right)+ \frac{1}{9}\left( fa6+fa7+fa8 \right)+ \frac{1}{6}\left( fa9+fa10 \right) \right)-1 \right] \cdot\frac{100}{3}$

$= \left[ \frac{1}{10}\left( \frac{10}{15}\left( fa1+fa2+fa3+fa4+fa5 \right)+ \frac{10}{9}\left( fa6+fa7+fa8 \right)+ \frac{10}{6}\left( fa9+fa10 \right) \right)-1 \right] \cdot\frac{100}{3}$

$= \left[ \frac{1}{10}\left( \frac{2}{3}\left( fa1+fa2+fa3+fa4+fa5 \right)+ \frac{10}{9}\left( fa6+fa7+fa8 \right)+ \frac{5}{3}\left( fa9+fa10 \right) \right)-1 \right] \cdot\frac{100}{3}$

Model M3: General Fatigue Index (first-order factor model: without domain separation)


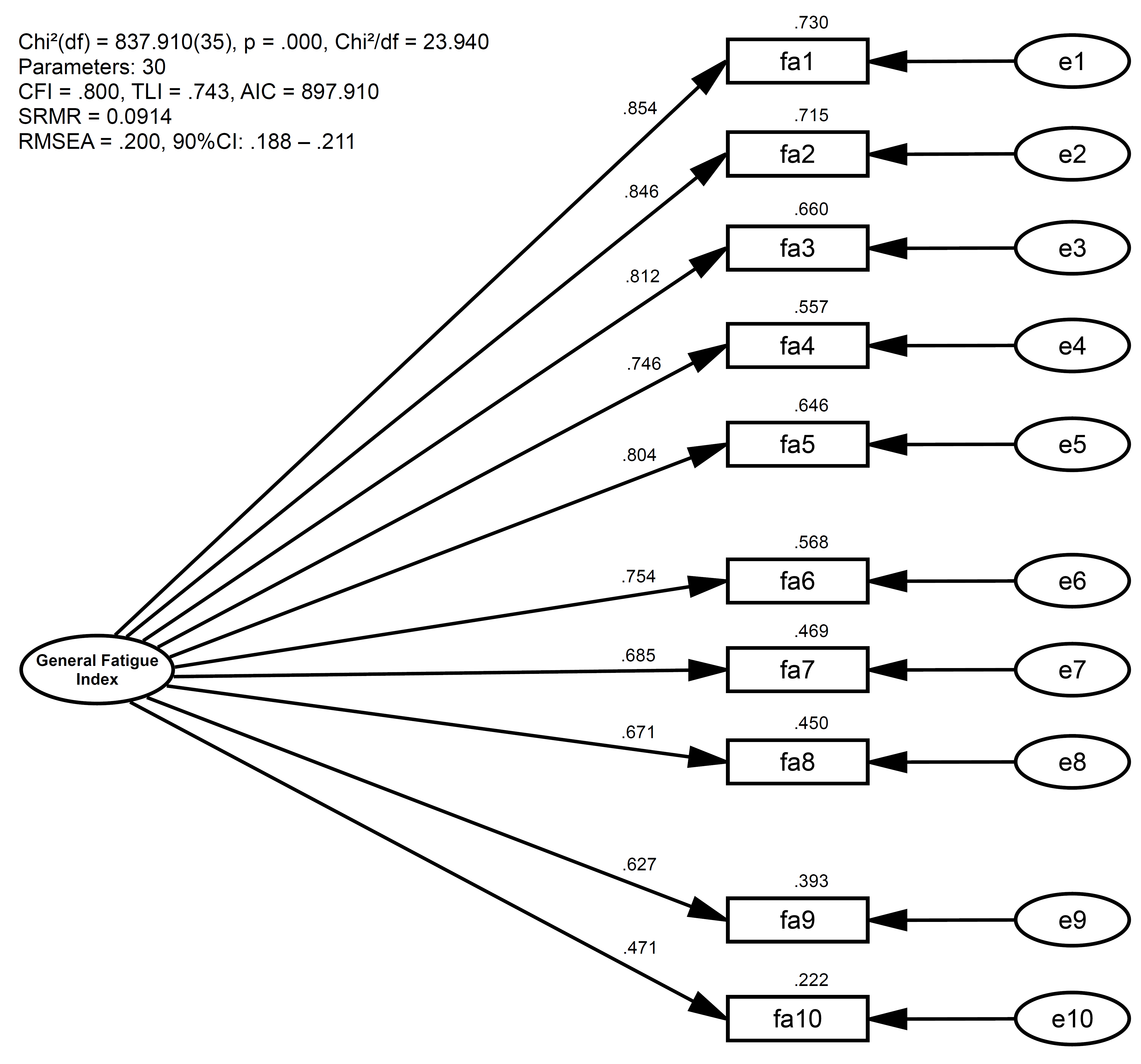


**Supplementary figure F2**: Model M3, first-order factor model, without criteria variables.

This model assumes that all fatigue items equally compose general fatigue, regardless of their corresponding type of fatigue.

**Scoring information**: The model implies the use of an overall score without the necessity of differentiating between the components to which the item normally would correspond. One could also say that it implies a weighted contribution of the components, according to their number of items. The more items a component has, the more important is its contribution to the common quality. The overall score is calculated using the following formula:

$$Overall score= \left( mean\left( all fatigue items \right)-1 \right) \cdot\frac{100}{3}$$

$$General fatigue index= \left[ \frac{1}{10}\left( fa1+fa2+fa3+fa4+fa5+fa6+fa7+fa8+a9+fa10 \right)-1 \right]\cdot\frac{100}{3}$$

**Example 3:** Consider again Patient A, who answered each physical item with 3 (= “quite a bit”) and all other items with 1 (=”not at all”), and patient B who answered all cognitive items with 3 and all other items with 1. This time both patients end up with different scores:

$$\boldsymbol{Patient A}= \left[ \frac{1}{10}\left( 3+3+3+3+3+1+1+1+1+1 \right)-1 \right]\cdot\frac{100}{3}=33.3$$

$$\boldsymbol{Patient B}= \left[ \frac{1}{10}\left( 1+1+1+1+1+1+1+1+3+3 \right)-1 \right]\cdot\frac{100}{3}=13.3$$

Note that M3 treats the *items* *equally*, but when using the domains (physical, emotional, and cognitive fatigue) *each domain has to be weighted* to obtain the same value:

General fatigue $= \left[ \left( \frac{1}{10}\left( fa1+fa2+fa3+fa4+fa5+fa6+fa7+fa8+fa9+fa10 \right) \right)-1 \right] \cdot\frac{100}{3}$

$= \left[ \left( \frac{1}{10}\left( fa1+fa2+fa3+fa4+fa5 \right)+\frac{1}{10}\left( fa6+fa7+fa8 \right)+\frac{1}{10}\left( fa9+fa10 \right) \right)-1 \right] \cdot\frac{100}{3}$

$= \left[ \left( \frac{1}{3}\cdot\frac{3}{10}\left( fa1+fa2+fa3+fa4+fa5 \right)+\frac{1}{3}\cdot\frac{3}{10}\left( fa6+fa7+fa8 \right)+\frac{1}{3}\cdot\frac{3}{10}\left( fa9+fa10 \right) \right)-1 \right] \cdot\frac{100}{3}$

$= \left[ \frac{1}{3}\left( \frac{3}{10}\left( fa1+fa2+fa3+fa4+fa5 \right)+ \frac{3}{10}\left( fa6+fa7+fa8 \right)+ \frac{3}{10}\left( fa9+fa10 \right) \right)-1 \right] \cdot\frac{100}{3}$

$= \left[ \frac{1}{3}\left( \frac{3}{2}\cdot\frac{1}{5}\left( fa1+fa2+fa3+fa4+fa5 \right)+ \frac{9}{10}\cdot\frac{1}{3}\left( fa6+fa7+fa8 \right)+ \frac{3}{5}\cdot\frac{1}{2}\left( fa9+fa10 \right) \right)-1 \right] \cdot\frac{100}{3}$

$= \left[ mean\left( \frac{3}{2}\cdot\frac{1}{5}\left( fa1+fa2+fa3+fa4+fa5 \right), \frac{9}{10}\cdot\frac{1}{3}\left( fa6+fa7+fa8 \right), \frac{3}{5}\cdot\frac{1}{2}\left( fa9+fa10 \right) \right)-1 \right] \cdot\frac{100}{3}$

$=\left[ mean\left( \frac{3}{2}\cdot mean\left( physical items \right), \frac{9}{10}\cdot mean\left( emotional items \right), \frac{3}{5}\cdot mean\left( cognitive items \right) \right)-1 \right] \cdot\frac{100}{3}$

Difference between the concepts of M2 and M3

As shown above, the imaginary patients in examples 2 and 3 seem to have the same burden of general fatigue when we use the concept of model M2. But when using the concept of M3 patient A with the physical tiredness is classified as more burdened than patient B with the cognitive burden. Only then, when patient B develops an additional burden (e.g. s/he circles at least 3 “quite a bit” on two more items of emotional and/ or physical fatigue) both would get the same score. Using any cut-off value this means that the concept of M3 gives preferential treatment to patients with physical fatigue above patients with emotional fatigue, followed by patients with cognitive fatigue.

These examples should illustrate that it makes a real difference in clinical practice, which concept is used.

Comments on the ROC analysis

Summary of the proposed criteria for diagnosis of CRF

To diagnose CRF with the proposed criteria, *first* a patient must have the feeling of fatigue, e.g. an unusually high or increased need to rest that has no relation to recent activities (symptom A1). *Second*, the patient additionally has to name *five out of ten symptoms*, which can be assigned to the three domains physical, emotional, and cognitive fatigue: five physical (A2, A5, A6, A9 and A11), three emotional (A4, A7, A8), and two cognitive symptoms (A3, A10). *Third*, all symptoms have to have presented at least nearly every day of the same two weeks within the past month. *Fourth*, they have to cause clinically significant distress or impairments in important areas of functioning. *Fifth*, they should evidently be the consequence of cancer or cancer therapy, and *sixth*, they should not primarily be the consequence of comorbid psychiatric disorders. (see [3] p. 374 table 2 or [4] p. 3386 table 1)

Constructing a binary reference standard for ROC analysis

Altogether, eleven symptoms are named in these criteria (A1 – A11). To all of them, except to symptom A11, we assign different self-rated items to indicate how many of these symptoms (except one) are met. In the end, to calculate the standard that indicates the target condition, we count the met criteria (A2 – A10) for all patients that fulfill the main criterion A1 (sum ranges from 0 to 9). A sum of at least four indicates the true positive condition, meaning that at least four out of nine criteria are met. Hence the negative condition indicates that five or more out of nine criteria have not been met. This means that the negative condition of our standard is more close to the negative condition of the proposed diagnostic criteria (positive: at least five out of ten symptoms are named, negative: five or more out of ten symptoms are not named), and it follows that we have a standard that identifies patients with a negative condition with more certainty than patients with a positive condition.

The items were chosen from the four instruments that were described in table 1 in section “Study measures” of the main document, (EORTC QLQ-C30, EORTC QLQ-FA13, HADS and SCNS SF-34). The first ten items in FA13 are excluded from this choice, because they are also used in FA12 and are necessary to compute the test (for assignment details see *supplementary table S1*).

Column “Formula” of supplementary table S1 shows how to calculate the value above which the patients are diagnosed with the respective symptom (A1 to A11, values: 1=yes/ true, 0=no/ false). The standard that indicates the approximated criterion for having CRF is then computed with *Standard=[A1*sum(A2, …, A11)≥4]* (see footnote a). The term *sum(A2, …, A11)* counts the fulfilled symptoms. If this sum is greater or even four, the patient has to fulfill the main criterion (= significant fatigue, answered by item *c10* with values greater or even three). Then s/he is labeled with the positive condition for a diagnostic interview regarding CRF (=approximated criterion for having CRF).

Justification for interpretation of ROC analysis results

To find the cut-off value at which the test indicates the positive condition of a patient, one has to find an optimal balance between sensitivity (SEN, true positive rate) and specificity (SPE, true negative rate). Normally one chooses the cutoff with the highest diagnostic ability shown by the maximum of the Youden Index [5]. This is the point where both measures (SEN and SPE) are highest in their sum, assuming they are equally important. However, for screening instruments, SEN should be prioritized over SPE if the instrument aims to detect new cases. SPE is more important, if the instrument aims to rule out the disease.

In our case, we have a standard that identifies patients with a negative condition with more certainty than it does patients with a positive condition. Therefore, and because we aim to identify patients who should be considered for a diagnostic interview, we optimize SEN at the expense of SPE. We choose the value greater than X as a cutoff that has at least a SEN of 90% and a SPE of more than 70%. This means that out of ten nominees for the diagnostic interview, we would miss one at most, and that out of ten no-nominees, no more than a maximum of three would have to undergo the interview. If there is more than one cutoff that fulfills this condition, we choose the one with the highest sum of SEN and SPE within the range presented.

Supplementary Table S1. Assignment details for approximating the standard based on the diagnostic criteria proposed by the Fatigue Coalition [3].

| **Symptoms** | | **Questionnaire** | **Item** | **Time-frame** | **Response for CN** | **Response for CP** | **Formula** ^a^ | **Frequency** ^b^ |
| --- | --- | --- | --- | --- | --- | --- | --- | --- |
| **Main** | **A1** | **Significant fatigue, diminished energy, or increased need to rest, disproportionate to any recent change in activity level.** | | | | | (c10≥3) | **N (%)** |
|  |  | Question: | ***Did you need to rest?*** | | | |  | 244 (45.8) |
|  |  | EORTC QLQ-C30 (subscale FA) | c10 | During the past week | ① not at all, ② a little | ③ quite a bit, ④ very much |  |  |
| **Physical** | **A2** | **Complaints about generalized weakness or limb heaviness** | | | | | (c12≥3) * (c3≥3) | 15 (2.8) |
|  |  | Questions: | ***Have you felt weak?*** | | | |  |  |
|  |  | AND | ***Do you have any trouble taking a short walk outside of the house?*** | | | |  |  |
|  |  | EORTC QLQ-C30 (subscale FA) | c12 | During the past week | ① not at all, ② a little | ③ quite a bit, ④ very much |  |  |
|  |  | AND | | | | |  |  |
|  |  | EORTC QLQ-C30 (subscale PF) | c3 | none | ① not at all, ② a little | ③ quite a bit, ④ very much |  |  |
|  | **A5** | **Insomnia or hypersomnia** | | | | | (c11≥3) | 192 (36.0) |
|  |  | Question: | ***Have you had trouble sleeping?*** | | | |  |  |
|  |  | EORTC QLQ-C30 (symptom-item IN) | c11 | During the past week | ① not at all, ② a little | ③ quite a bit, ④ very much |  |  |
|  | **A6** | **Experience of sleep as unrefreshing or nonrestorative (Non-restorative sleep)** | | | | | (c18≥3) | 289 (54.2) |
|  |  | Question: | ***Were you tired?*** | | | |  |  |
|  |  | EORTC QLQ-C30 (subscale FA) | c18 | During the past week | ① not at all, ② a little | ③ quite a bit, ④ very much |  |  |
|  | **A9** | **Difficulty completing daily tasks attributing to feeling fatigued** | | | | | (fa13_11≥3) * (fa12_11≥3) | 70 (33.2) |
|  |  | Questions: | ***Did you have trouble completing things?*** | | | |  |  |
|  |  | AND | ***Did tiredness interfere with your daily activities (home, leisure activities)*** | | | |  |  |
|  |  | EORTC QLQ-FA13 | fa13_11 ^c^ | During the past week | ① not at all, ② a little | ③ quite a bit, ④ very much |  |  |
|  |  | AND | | | | |  |  |
|  |  | EORTC QLQ-FA12 (criteria item) | fa12_11 ^d^ | During the past week | ① not at all, ② a little | ③ quite a bit, ④ very much |  |  |
|  | **A11** | **Post-exertional malaise lasting several hours** | | | | | 0 | - |
|  |  | no item(s) assigned |  |  |  |  |  |  |
| **Emotional** | **A4** | **Decreased motivation or interest to engage in usual activities** | | | | | (c24≥3) | **N (%)** |
|  |  | Question: | ***Did you feel depressed?*** ^e^ | | | |  | 177 (33.2) |
|  |  | EORTC QLQ-C30 (subscale EF) | c24 | During the past week | ① not at all, ② a little | ③ quite a bit, ④ very much |  |  |
|  | **A7** | **Perceived need to struggle to overcome inactivity** | | | | | (s2≥5) * (ha6≤2) | 6 (1.1) |
|  |  | Questions: | ***Lack of energy / tiredness.*** | | | |  |  |
|  |  | AND | ***I feel restless as if I have to be on the move.*** | | | |  |  |
|  |  | SCNS SF-34 (subscale PN) | s2 | Last month | ① no problem,  ② already supported,  ③ low need,  ④ moderate need | ⑤ high need |  |  |
|  |  | AND | | | | |  |  |
|  |  | HADS (subscale ANX) | ha6 | During the past week | ③ not very much, ④ not at all | ① very much indeed, ② quite a lot |  |  |
|  | **A8** | **Marked emotional reactivity (e.g. sadness, frustration or irritability) to feeling fatigued** | | | | | (ha1≤2) | 155 (29.1) |
|  |  | Question: | ***I feel tense or 'wound up.*** | | | |  |  |
|  |  | HADS (subscale ANX) | ha1 | During the past week | ③ time to time, occasionally,  ④ not at all | ① most of the time,  ② a lot of the time |  |  |
| **Cognitive** | **A3** | **Diminished concentration or attention** | | | | | (c20≥3) | 131 (24.6) |
|  |  | Question: | ***Have you had difficulty in concentrating on things, like reading a newspaper or watching television?*** | | | |  |  |
|  |  | EORTC QLQ-C30 (subscale CF) | c20 | During the past week | ① not at all, ② a little | ③ quite a bit, ④ very much |  |  |
|  | **A10** | **Perceived problems with short-term memory** | | | | | (c25≥3) | 120 (22.5) |
|  |  | Question: | ***Have you had difficulty remembering things?*** | | | |  |  |
|  |  | EORTC QLQ-C30 (subscale CF) | c25 | During the past week | ① not at all, ② a little | ③ quite a bit, ④ very much |  |  |

**Abbreviations**: CN=negative condition, CP=positive condition, FA=fatigue, PF=physical functioning, CF=cognitive functioning, EF=emotional functioning, IN=insomnia, ANX=anxiety, PN= physical and daily living needs.
**^a^** Standard=[A1*sum(A2, …, A11)≥4]; **^b^** frequency of the approximated symptom; **^c^** Former item 11 of FA13, dropped in FA12; **^d^** Former item 12 of FA13, now labeled as item 11 in FA12; **^e^** The German version uses the word “dejected” instead of “depressed”.

Supplementary table S2. Item wordings for the EORTC QLQ-FA12 module for CRF (adapted from Weis et al. [1], Supplementary Table 1).

| **Physical fatigue** | |
| --- | --- |
| fa1 | Have you lacked energy? |
| fa2 | Have you felt exhausted? |
| fa3 | Have you felt slowed down? |
| fa4 | Did you feel sleepy during the day? |
| fa5 | Did you have trouble getting things started? |
| **Emotional fatigue** | |
| fa6 | Did you feel discouraged? |
| fa7 | Did you feel helpless? |
| fa8 | Did you feel frustrated? |
| **Cognitive fatigue** | |
| fa9 | Did you have trouble thinking clearly? |
| fa10 | Did you feel confused? |
| **Criteria variable for interference with daily activities** (content of role functioning) | |
| fa11 | Did tiredness interfere with your daily activities (home, leisure activities)? |
| **Citeria variable for interference with social life** (content of social functioning) | |
| fa12 | Did you feel that your tiredness is (was) not understood by the people close to you? |

The unidirectional items are ranging from 1 (not at all) to 4 (very much), higher values represent higher levels of fatigue during the past week.

References

1. Weis J, Tomaszewski KA, Hammerlid E, Arraras JI, Conroy T, Lanceley A, et al. International Psychometric Validation of an EORTC Quality of Life Module Measuring Cancer Related Fatigue (EORTC QLQ-FA12). J Natl Cancer Inst. 2017;109:1–8. doi:10.1093/jnci/djw273.

2. Fayers PM, Aaronson NK, Bjordal K, Groenvold M, Curran D, Bottomley, A on behalf of the EORTC Quality of Life Group. The EORTC QLQ-C30 Scoring Manual (3rd Edition). Brussels; 2001.

3. Cella D, Peterman A, Passik S, Jacobsen P, Breitbart W. Progress toward guidelines for the management of fatigue. Oncology (Williston Park, N.Y.). 1998;12:369–77.

4. Cella D, Davis K, Breitbart W, Curt G, Fatigue Coalition. Cancer-related fatigue: prevalence of proposed diagnostic criteria in a United States sample of cancer survivors. J Clin Oncol. 2001;19:3385–91.

5. Youden WJ. Index for rating diagnostic tests. Cancer. 1950;3:32–5. doi:10.1002/1097-0142(1950)3:1<32::AID-CNCR2820030106>3.0.CO;2-3.
